# Dietary Intake, Body Composition, and Nutritional Knowledge of Elite Handball Players

**DOI:** 10.3390/nu16162773

**Published:** 2024-08-20

**Authors:** Giannis Arnaoutis, Maria Alepoudea, Konstantinos D. Tambalis, Labros S. Sidossis

**Affiliations:** 1 School of Health Science and Education, Department of Nutrition and Dietetics, Harokopio University, El. Venizelou Ave. 70, 17671 Athens, Greece; 2 School of Physical Education and Sports Science, National and Kapodistrian University of Athens, Daphne, 17237 Athens, Greece; ktambal@phed.uoa.gr; 3 Department of Kinesiology and Health, Rutgers University, New Brunswick, NJ 08901, USA; lsidossis@kines.rutgers.edu

**Keywords:** nutritional behavior, sports nutrition, elite athletes, nutrition questionnaire, dietary consumption, macronutrients, athletic performance

## Abstract

Nutrition affects both body composition and, consequently, athletic performance. Only a few studies have assessed the nutritional behavior and knowledge of elite players. The present study aimed to assess the dietary intake, body composition, and nutritional knowledge of elite handball players. Thirty-nine handball players (age: 23.2 ± 2.7 years, weight: 88.2 ± 10.1 kg, height: 1.87 ± 0.07 m, and years of training: 13 ± 2) participated in the study. The athletes completed a set of anthropometric measurements, a 24 h food recall, and a translated edition of Abridged Nutrition for Sport Knowledge Questionnaire (A-NSKQ). The average body fat percentage was 16.7 ± 3.8%, while the average fat free mass was 73.9 ± 8.5 kg. The athletes’ average daily energy intake was 2606.6 ± 756 kcal, while the average daily intake for carbohydrates, proteins, and fats was 243.85 ± 107.79 g [2.8 ± 1.3 g/kg BW/d—37.2 ± 10.5% of Total Energy Intake (TEI)], 131.59 ± 53.28 g (1.51 ± 0.7 g/kg BW/d—20.3 ± 6.9% of TEI), and 117.65 ± 40.52 g (40.9 ± 9.9% of TEI), respectively. For iron, calcium, and vitamin D, the average daily intakes were 19.33 ± 10.22 mg, 1287.7 ± 676.42 mg, and 3.22 ± 3.57 mcg respectively. The average success rate on the A-NSKQ was only 38.5 ± 10.7% out of 100. Elite handball players exhibit inadequate dietary intake and sports nutrition knowledge. Nutritional education should be a primary concern towards the amelioration of their athletic performance.

## 1. Introduction

Handball is a professional and energy-demanding Olympic team sport characterized by repeated bouts of high-intensity explosive offensive and defensive actions (jumping and sprinting), handball-specific movements (throwing, catching, and blocking) and changes in direction primarily fueled through anaerobic lactic–alactic metabolism, followed by short phases of low-intensity jogging, along with frequent body contacts between the players of the two opponent teams [1]. In order for professional handball players to reach a high performance, they need to develop and maximize skills such as muscle strength, agility, speed, and endurance [2]. The high physiological demands of this sport can be concluded by the data deriving from a recent and comprehensive review for elite handball players [3]. The findings indicate that an elite handball player covers an average of 3664.4 ± 1121.6 m during a match, with a running pace of 84.8 ± 17.2 m∙min^−1^ on average. As far as the internal load is concerned, the average heart rate ranges of the athlete ranges from 70 ± 11.0% to 90.1 ± 4.3% of the heart rate maximum (%HRmax), whereas the mean blood lactate concentration (BLC) ranges from 3.6 ± 2.1 to 4.8 ± 1.9 mmol·L^−1^, exceeding 8.0 mmol·L^−1^ at some points during the match,.

An important parameter for maximizing sports performance is nutrition. Dietary intake is a modifiable parameter that can contribute positively to the amelioration of athletic performance since it is directly correlated to optimal body function and composition [4]. Nutritional strategies that define the type, amount, and timing of intake of food, fluids, and supplements are proven to enhance athletic performance by allowing the athlete to stay healthy and injury-free, while optimizing, at the same time, both the functional and the metabolic adaptations to periodized training programs or official athletic events [5]. In indoor team sports, carbohydrates represent the most important fuel since glucose availability is the limiting factor for both physical and cognitive performance. The current recommendations are in the range of 6–10 g/kg of body weight for training lasting 1–3 h which is the most common duration for handball training [6,7]. Additionally, the high-intensity intermittent nature of handball, which involves acceleration, deceleration, and changes in direction, all of the above implicating eccentric and, consequently, muscle-damaging contraction, along with frequent bodily contact, highlights the importance of muscle restoration through the provision of an adequate amount of protein. The general established recommendations of protein for athletes range between 1.2 and 2 g/kg of body weight per day [7]. Fat, on the other hand, does not represent an important energy source in high-intensity intermittent sports, since it cannot act as a substrate for the development of these explosive efforts. On the contrary, high fat consumption can be counterproductive for an athlete in terms of body composition and slow gastric emptying. Although fat intake recommendations have not been established for the athletic population, it is suggested that athletes should not exceed the established 25–30% fat recommendations of total daily energy [8]. Elite athletes commonly consume sports supplements to enhance athletic performance. In a study examining the use of nutritional supplements in competitive handball players, 67.1% of professional players reported the use of at least one supplement, with the most prevalent being sports drinks (42.2%), energy bars (35.3%), caffeine-containing products (31.6%), and creatine (22.3%) [9].

Sports nutrition knowledge can be defined as the understanding of the nutritional factors that influence training, athletic performance, and recovery. This specialized knowledge overcomes general nutrition, which typically covers food groups, nutrient sources, and overall health, by addressing the unique needs of high-performing athletes [10]. Accordingly, nutritional knowledge, one of the few adaptable determinants of dietary behaviors, has been positively correlated with dietary intake [11,12]. However, many of the studies applied to the sports population indicate that the majority of the athletes appear to have low nutrition knowledge, putting them at risk of inappropriate dietary choices that could decrease the ability to optimally perform and increase the risk of injury [13]. Indicatively, 331 collegiate athletes reported low sports nutrition knowledge [14], while only 55.2% of Division III football players scored correctly in a nutrition knowledge quiz [15]. More precisely, in a study with professional handball players, macronutrient intakes as a percentage of total energy intake were below the recommended allowances for carbohydrates, and above the recommended allowances for fats [2]. The aforementioned results are in accordance with those from a recent systematic literature review which indicated that professional and semi-professional athletes’ dietary intakes exceed the recommendations during training and competition for protein and/or fat but are lower for total energy intake and carbohydrate consumption [16]. Hence, there is a need for more studies examining the nutritional knowledge and behavior of professional athletes, and there is a parallel lack of studies examining these parameters in elite athletes competing at the international level [17].

In the last few years, there has been a rise in scientific and research interest in quantifying the nutritional knowledge of sports populations since it has been acknowledged as one of the few modifiable factors that determine dietary behavior [18]. At the same time, the tools that have been used so far for assessing the level of sports nutritional knowledge are poorly evaluated [12]. Stronger relations between nutritional knowledge and intake in athletes may not appear also because the link between them is influenced by factors such as taste; food preferences; cultural, religious, or family beliefs; and skills in shopping, food preparations, and label reading [4,10]. All these factors make the assessment more complicated and difficult to be accurately defined [11,16]. Despite the effort that is being made to develop validated evaluation tools that can sufficiently assess the level of nutritional knowledge in athletic populations, there is still much work to be carried out specifically to acquire reliable data from multiple athletic fields.

Therefore, the aim of this study was to investigate the dietary intake and nutritional knowledge of professional Greek handball players preparing for the European Championship.

## 2. Materials and Methods

### 2.1. Subjects

Thirty-nine male professional Greek handball players were recruited to participate in the study. Their physical characteristics are presented in Table 1. All participants were highly trained athletes who had competed in national and international championships throughout their careers. Each participant gave written informed consent, and the protocol was approved by the institutional review board. The study was carried out in accordance with the Declaration of Helsinki (1983) of the World Medical Association. Eligibility criteria for participation in the study included a normal physical examination and the absence of any metabolic, cardiovascular, or renal disease.

### 2.2. Study Organization

During their stay in a national team selection training camp before the European championship, the athletes agreed to participate in an assessment of their anthropometric characteristics, nutritional intake, and nutritional knowledge.

### 2.3. Anthropometric Measurements

Athletes’ body mass and stature were measured in the morning, before training and after they had their bladder emptied, using a standardized procedure. Body mass was measured without shoes, in the standing upright position and recorded on a sensitive digital scale with a precision of ±0.01 kg (Seca, model: 7701321004; Vogel & Hamburg, Hamburg, Germany). Height was measured using a stadiometer (Leicester portal height measure, Tanita HR 001, Tokyo, Japan) to the nearest 0.1 cm with the athletes’ weight equally distributed on their feet and their head, back, and buttocks on the vertical line of the height gauge. Body mass index (BMI) was consequently calculated as body weight in kilograms divided by the square of height in meters (kg/m^2^) based on the aforementioned measurements.

### 2.4. Body Composition Assessment

Body composition was evaluated via a Lange skinfold caliber (Model 68902, Cambridge Scientific Industries, Inc, Cambridge, MD, USA) using male 7-site skinfolds (triceps, chest, midaxillary, subscapular, abdomen, suprailiac, and thigh) taken on the right side of the body. Each skinfold measurement was performed twice at each site, and only if the difference exceeded 2 mm was a third measurement performed. The average of two measurements within 2 mm was recorded. The recorded measurements were then used to estimate the fat mass (kg), fat-free mass (kg), and body fat percentage (%), with the use of the equations by Jackson and Pollock [19]. Fat-free mass (FFM) was then used to estimate resting metabolic rate (eRMR, kcals) using the Cunningham Equation: resting metabolic rate = 500 + 22 (FFM) [20]. Subsequently, the eRMR was multiplied by an activity factor of 1.8 in order to calculate the estimated total daily energy expenditure (eTEE). All anthropometric measurements were performed by a trained physical education professional, and a standardized procedure was implemented, along with a systematic calibration of the devices (e.g., weight scales and skinfold caliber), in order to ensure maximum validity and accuracy.

### 2.5. Nutritional Assessment

Nutritional intake was measured via a 24 h dietary recall conducted on a typical training day within a week. More precisely, data on food intakes were obtained during individual interviews to request information from each participant about the types of foods and serving sizes. Recall accuracy was facilitated with a set of photographs of the prepared foods and dishes that the athletes consumed during their stay from the camp’s food buffet. The Nutritionist Pro^®^ software program was used to convert food intakes to absolute and percentage values of recommended intakes of each nutrient for individual athletes.

Nutritional knowledge was assessed via a translated version of the Abridged version of the Nutrition for Sport Knowledge Questionnaire (A-NSKQ) including the most recent modifications [21,22]. Briefly, the A-NSKQ is a valid and reliable brief questionnaire designed to assess general nutrition knowledge (GNK) and sports nutrition knowledge (SNK) including 17 and 20 questions, respectively, based on current sports nutrition recommendations [5,23,24]. More precisely, the first sub-section (‘general nutrition knowledge’) includes questions that assess knowledge of energy density; the role and sources of macro- and micronutrients; and alcohol-related consumption issues. The sports nutrition knowledge sub-section includes 20 questions that assess knowledge of athletes’ macronutrient and fluid requirements; weight loss and muscle gain techniques for athletes; and supplement-related issues. The A-NSKQ has been shown to exhibit high construct validity (*p* < 0.001) with good test-to-test concordance (r = 0.80; *p* < 0.001) among athletes. From the original study, scores > 47% represent greater than average nutrition knowledge. The above assessments were conducted by a trained and experienced registered dietician.

### 2.6. Statistical Analyses

The data are reported with descriptive statistics. For numerical variables, the arithmetic means and standard deviation of the mean were used. The results for categorical variables are reported as percentage frequencies. To determine whether the data fitted a parametric model, the Kolmogorov–Smirnov test was used to verify normal distribution. For the current study, athlete’s nutrition knowledge (average or greater), self-rated nutrition knowledge categories (poor or good), and total energy consumption (low, recommended, or high) were calculated. The chi-square test evaluated associations between the categorical variables. Comparisons of the participant’s compliance with the recommendations for protein, carbohydrates, fat, and total energy consumption between those with average nutrition knowledge and those with greater knowledge were performed using the independent samples *t*-test, after testing for equality of variances using the Levene test. Furthermore, linear regression analysis was applied to examine the association of various potential predictors (i.e., age, BMI, waist circumference, total fat, vitamins, and micronutrients) with total energy consumption. The results from the regression models are presented as beta coefficients. The normality of the residuals was graphically assessed through P-P plots of standardized residuals. Collinearity was tested using the VIF criterion (values > 4 indicate the presence of collinearity and the variable was excluded from the model). The assumptions of linearity for the continuous independent variables and constant variance of the standardized residuals were assessed by plotting the residuals against the fitted values. Moreover, aiming to investigate the potential effect of several variables (e.g., age, BMI, total fat, and vitamins and micronutrients) on the category (recommended or high) of total energy consumption, binary logistic regression analysis with Enter as the selected method was implemented, and OR with the corresponding 95% CI were calculated to obtain adjusted association of covariates while controlling for confounding. The distribution of the independent variables in the models was carried out with scientific criteria. Hosmer and Lemeshow’s goodness-of-fit test was calculated to evaluate the model’s goodness of fit and residual analysis was implicated using the dbeta, the leverage, and Cook’s distance D statistics to identify outliers and influential observations. All statistical analyses were performed using the SPSS version 23.0 software for Windows (SPSS Inc. Chicago, II, USA). The statistical significance level from two-sided hypotheses was set at *p*-value < 0.05.

## 3. Results

The results for the energy and macronutrient intakes as a percentage of the allowances for athletes recommended by the Academy of Nutrition and Dietetics, Dietitians of Canada (DC), American College of Sports Medicine (ACSM), and the International Society of Sports Nutrition [5,7] are shown in Table 2.

The total daily energy intake and daily carbohydrate consumption were below the recommended amount. The protein intake, on the other hand, met the suggested amount, while, in contrast, the fat intake was above the recommended amount.

The analysis about the Recommended Dietary Allowance (RDA) or Daily Adequate Intake (AI) from the National Academy of Medicine for micronutrients is presented in Table 3. Indicatively, this showed that the athletes consumed adequate amounts of micronutrients of key interest for an athlete like iron and calcium, while, on the contrary, vitamin D levels were well below the suggested ones.

**Figure 1:**
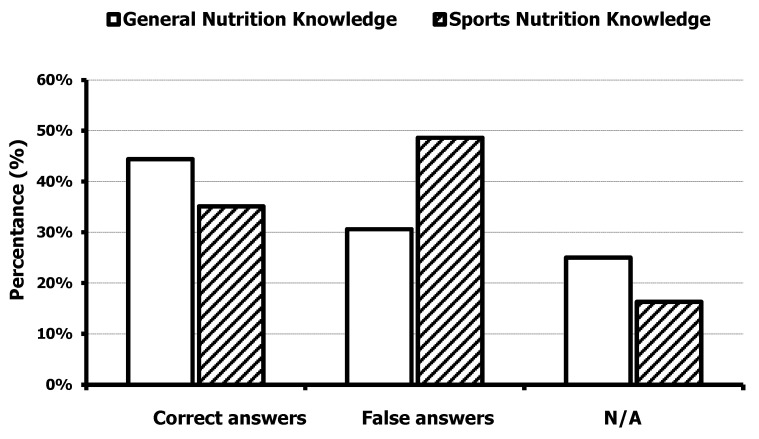
Results from the general nutrition and from the sports nutrition knowledge sub-sections of the Abridged version of the Nutrition for Sport Knowledge Questionnaire (A-NSKQ).

The athletes scored correctly on the A-NSKQ with a success rate of only 38.54 ± 10.7% out of 100. The correct vs. wrong answers for the general knowledge sub-section of the A-NSKQ was 44 ± 11.6 vs. 30.4 ± 8.6%, while, for the sports nutrition knowledge sub-section, it was 35.1 ± 13.3 vs. 48.5 ± 10.2%, respectively (Figure 1). These results indicate that athletes had low knowledge overall, but better general vs. sports nutrition knowledge. The most commonly missed questions (>50% answered incorrectly) were those relating to acceptable macronutrient and micronutrient distribution ranges for athletes, and macronutrient function. Over 75% of participants correctly answered questions related to the efficacy of specific sport supplements and recommended strategies in order to achieve muscle gains.

Moreover, the chi-squared analysis between athletes’ nutrition knowledge categories (average or greater) and self-rated nutrition knowledge categories (poor or good) did not reveal significant differences (*p* > 0.05). Likewise, the same analysis did not show statistically significant differences between nutrition knowledge and energy consumption categories (low, recommended, or high). Those athletes who incorporated greater nutrition knowledge did not present significant differences in compliance with the recommendations for protein, carbohydrates, fat, and total energy consumption compared to those with average knowledge (all *p*-values > 0.05).

Table 4 presents the beta weights of athletes’ anthropometric measurements, vitamins, and micronutrient consumption for total energy consumption, suggesting that only age (−0.48) and BMI (0.68) contribute significantly to predicting an athlete’s energy consumption.

Finally, to assess the probable associations of the aforementioned factors on the recommended vs. high total energy consumption, stepwise logistic regression analyses were conducted. No statistically significant results were incorporated.

## 4. Discussion

In the present study, we examined the dietary intake and the nutritional knowledge of elite Greek handball players during their stay in a training camp a few weeks before the European Championship. The main findings of the study were that the athletes underestimated their total energy intake and especially the intake coming from sources high in carbohydrates, consuming more fat than the recommended amount, and, at the same time, that they exhibited poor knowledge in general, as well as in sports nutrition.

In general, the handball players in the present study failed to meet the recommendations for nutrient intake. The energy intake was about 1200 kcal/day lower than the eTEE. The results deriving from the dietary intake analysis in the present study are in line with those from a descriptive feasibility study in which female volleyball players had a lower energy and carbohydrate intake than the recommendations pre-season [26], as well as with those from professional wrestlers in which the athletes lacked about 1200 kcal/day and consumed much less carbohydrates (3.1 g/kg) [27]. Accordingly, in a longitudinal study of 14 handball players, the energy intake was consistently below the recommended allowances. Macronutrient intakes as a percentage of total energy intake were also below the recommended allowances for carbohydrates, and above the recommended ones for fats [2], exhibiting comparable results with the present study. Although it is well-documented that carbohydrates are the primary fuel for team sports, with the recommendations ranging from 6 to 10 g/kg, in the present study, the athletes were below the recommended low range (6 g/kg), a finding that it is also highlighted in a recent systematic review [6]. Overall, a total energy and macronutrient imbalance is constantly present in most studies dealing with team sport athletes [16,28,29,30], with most athletes reporting consuming diets high in protein and fat, at the expense of carbohydrates [6]. These discrepancies highlight the need for targeted educational interventions to ensure that all players have access to accurate and up-to-date nutritional information.

It is well-known that exercise stresses many metabolic pathways in which micronutrients are necessary, and training results in muscle biochemical adaptations that increase the need for some of these micronutrients. As far as the assessment of micronutrient intake in elite team sport athletes is concerned, there is a lack of studies, and, to our knowledge, only the study from Molina and his colleagues found similar results with ours, emphasizing the low consumption of sources high in vitamin D [2]. It is known that vitamin D regulates calcium and phosphorus absorption and metabolism and plays a key role in maintaining bone health. Likewise, multiple studies indicate its beneficial impacts on physical fitness, the integrity of the skeletal structure, and the muscular strength of the lower and upper extremities in athletes [31,32]. On the contrary, handball players train and compete almost exclusively indoors and, therefore, are at a higher risk of developing vitamin D deficiency and, consequently, observing decrements in their performance.

Thus, the accurate assessment and adequate consumption of macro- and micronutrients can help athletes ameliorate their health, training adaptations, and performance.

Nutrition knowledge is a vital factor for athletes in order to ameliorate their sport performance, support their post-training recovery, manage their body composition, and, ultimately, maintain health. However, despite the difficulties in assessing it through validated tools, the results indicate that most athletes show a significant lack of nutrition knowledge. In the present study, only 44% of the handball players answered correctly in the general nutrition knowledge assessment, while the percentage of correct answers in the sports nutrition knowledge assessment barely reached 35%. The percentage of poor nutrition knowledge was much higher in a large sample of Jordanian athletes (n = 3323), with 88.3% of the participants scoring below 50% [33]. The average score on the Nutrition Sports Knowledge Questionnaire was 45 ± 9.6% among collegiate athletes [26] and 36.9 ± 19.1% among National Collegiate Athletic Association (NCAA) Division III (DIII) athletes [14]. Slightly higher was the mean score for NCAA DIII football players, reaching 55.2 ± 16.3% [15], while elite ultra-marathon runners reached a 77.5 ± 16.9% nutritional knowledge score [12]. Finally, in accordance with the aforementioned results, a study involving a high number of elite team sport athletes clearly indicated that the athletes need to significantly improve their nutrition knowledge, especially in the areas of macronutrients and micronutrients [10]. These results might indicate a potential link between the level of the athletes and their knowledge about sports nutrition, which warrants further research.

In more detail, in our study, athletes correctly answered a smaller percentage of questions in the sports-nutrition-specific section of the questionnaire compared to the general nutrition knowledge section. This suggests that the application of nutrition knowledge within the context of sports performance should be an area of focus for athletes. This lack of sports nutrition knowledge among athletes may also be linked to the quality of information and education that they are receiving from their coaches and support staff [34], since studies that have examined the sports nutrition knowledge of various professionals involved with team sports found that only 35.9% of coaches had sufficient nutritional knowledge [34,35]. Therefore, it is highly suggested that nutritional education programs should thoroughly be extended to coaches and other individuals working with the athletes systematically.

In overall, integrating nutrition education into the training programs of teams, which, in turn, will allow players to make correct decisions about their dietary intake and, consequently, avoid dietary choices that could compromise their performance and recovery, seems to be a necessity and should be prioritized.

Finally, as far as the results from the body composition analysis are concerned, the percentage of body fat was measured higher in the present study (16.7 ± 3.8%) compared to a small number of similar studies performed within the same athletic population in which the percentage of body fat was measured, 11.6 ± 2.5% [2], 11.2 ± 4.6% [36], and 15.4 ± 3.7% [37]. These data should concern the participants of the present study, since the nature of handball requires explosiveness and agility, which, in turn, are characteristics deriving from increased muscle mass, and, at the same time, they should understand the importance of a long-term nutritional and body composition assessment with prompt interventions. However, more research in this area is clearly warranted.

The strengths of this study are the level of the athletes that participated along with their dietary analysis, since there is a lack of studies involving professional athletes in-season, preparing for an important tournament [17], and the fact that the data were collected during real-life training settings [38]. Furthermore, the present study has clear practical implications, since, by addressing key research questions related to the nutritional knowledge and dietary practices of handball players, it offers practical insights that can develop evidence-based nutrition education programs tailored to the specific needs of athletes in this sport.

A limitation of the study is the method used to assess the dietary intake and the fact that only a one-day food recall was applied. The reliance on self-reported data commonly introduces response bias and inaccuracies. Participants may overestimate and/or underestimate their level or adherence to recommended dietary guidelines. Unfortunately, due to the importance of the preparation for the following Championship, the researchers were not allowed to conduct more assessments. The latter was also the reason why blood samples were not collected, and, consequently, why we were not able to carry out a more precise assessment through the interpretation of multiple biochemical indices. Moreover, the study did not consider multiple external factors that influence the nutritional knowledge and dietary practices among handball players, such as socio-economic status, access to resources, and cultural influences [16]. The failure to address these factors limits the study’s ability to provide detailed insights into the complex interaction between nutrition and athletic performance.

For future research, it is suggested that we also conduct the dietary assessment on the weekend since it can provide researchers with additional data. Finally, it is a common observation that there is a lack of studies using a female athletic population. Therefore, it is highly encouraged that forthcoming studies thoroughly investigate this very interesting and under-studied population.

## 5. Conclusions

In conclusion, the findings of the present study highlight the importance of nutritional knowledge in optimizing the nutritional intake and, consequently, the performance of elite handball players. By addressing the numerous gaps in nutritional knowledge, providing targeted education, and promoting collaboration between athletes, coaches, and sports nutritionists, teams can empower their players to make informed dietary choices that support their athletic goals. Future research should explore the effectiveness of educational interventions in improving nutritional knowledge and dietary practices among athletes, with the ultimate aim of enhancing their athletic performance and overall health.

## Figures and Tables

**Table 1:** Physiological characteristics of the study participants.

Characteristics	*n* = 39
Age (years)	23.2 ± 2.7
Years of training	13 ± 2
Weight (kg)	88.2 ± 10.1
Height (m)	1.87 ± 0.07
BMI (kg∙m^−2^)	25.2 ± 2.1
Body fat (%)	16.7 ± 3.8
Fat mass (kg)	14.6 ± 4.4
Fat-free mass (kg)	73.9 ± 8.5
eRMR (kcal)	2.125 ± 277
eTEE (kcal)	3.825 ± 498

Values are means ± SD. eRMR. estimated resting metabolic rate. eTEE, estimated total energy expenditure. BMI, body mass index.

**Table 2:** Daily energy intake and macronutrient consumption.

Variable	Mean ± SD	Recommended Intake [5,7]
Energy (kcal/d)	2606 ± 756	
Energy (kcal/kg BW/d)	29.37 ± 8.5	37–41
Carbohydrates		
Per day (g/d)	243.85 ± 107.79	
Per unit of body mass (g/kg BW/d)	2.78 ± 1.3	6–10
% total energy intake	37.23 ± 10.5	
Protein		
Per day (g/d)	131.59 ± 53.28	
Per unit of body mass (g/kg BW/d)	1.51 ± 0.7	1.2–2.0
% total energy intake	20.27 ± 6.9	
Fat		
Per day (g/d)	117.65 ± 40.52	
Per unit of body mass (g/kg BW/d)	1.34 ± 0.4	0.9–1.1
% total energy intake	40.98 ± 9.9	

Values are means ± SD.

**Table 3:** Daily micronutrient intake.

Micronutrient	Mean ± SD	Recommended Intake [5,25]
Iron (mg)	19.3 ± 10.2	8
Calcium (mg)	1287.7 ± 676.4	1000
Vitamin D (mcg)	3.2 ± 3.5	15
Vitamin A (mcg)	1077.5 ± 2921	900
Vitamin E (mg)	8.1 ± 12.0	15
Vitamin C (mg)	116.7 ± 97.2	90
Thiamin (mg)	2.4 ± 1.6	1.2
Riboflavin (mg)	3.2 ± 4.1	1.3
Niacin (mg)	19.1 ± 15.9	16
Vitamin B6 (mg)	2.3 ± 1.4	1.3
Biotin (mcg)	47.9 ± 95.6	30
Vitamin B12 (mcg)	12.4 ± 46.2	2.4
Magnesium (mg)	293.2 ± 159.4	400
Selenium (mcg)	85.9 ± 56.4	55
Sodium (mg)	3454.8 ± 1731.4	1500
Potassium (mg)	2877.8 ± 1026.9	3000
Folate (mcg)	387.9 ± 291.5	400

Values are means ± SD.

**Table 4:** Multivariate regression models were used to evaluate the association of participant’s anthropometric factors and vitamin and micronutrient consumption with total energy consumption.

Predictors	Model 1 Anthropometric Factors Beta	Model 2 Anthropometric Factors + Vitamins Beta	Model 3 Anthropometric Factors + Vitamins + Micronutrients Beta
Men			
Age (year)	−0.48 *	−0.48 *	−0.48 *
Waist circumference (cm)	−0.03	−0.02	−0.02
Fat-free mass (kg)	0.48 *	0.47 *	0.47 *
Body fat (kg)	−0.24	−0.23	−0.23
Vitamin A (mg)		0.37	0.37
Vitamin D (mcg)		0.10	0.10
Vitamin E (mg)		−0.19	−0.19
Vitamin C (mg)		0.02	0.02
Vitamin B1 (mg)		−0.13	−0.13
Vitamin B2 (mg)		−0.01	−0.01
Vitamin B3 (mg)		0.42	0.42
Vitamin B6 (mg)		0.45	0.45
Vitamin B12 (mcg)		−0.26	−0.26
Folic acid (mcg)		−0.24	−0.24
Biotin (mcg)		0.16	0.16
Ca (mg)			0.14
Mg (mg)			0.14
Fe (mg)			0.22
Se (mcg)			0.16
Na (mg)			0.28
K (mg)			−0.08

Model 1: Age and body mass index and waist circumference and body fat; Model 2: Model 1 + vitamins; Model 3: Model 2 + micronutrients. * *p*-value < 0.05

## Data Availability

The data presented in this study are available for non-commercial scientific inquiry and educational use upon request from the corresponding author.

## References

[1] Cunniffe B., Fallan C., Yau A., Evans G.H., Cardinale M. (2015). Assessment of Physical Demands and Fluid Balance in Elite Female Handball Players During a 6-Day Competitive Tournament. Int. J. Sport. Nutr. Exerc. Metab..

[2] Molina-López J., Molina J.M., Chirosa L.J., Florea D., Sáez L., Jiménez J., Planells P., Pérez de la Cruz A., Planells E. (2013). Implementation of a Nutrition Education Program in a Handball Team; Consequences on Nutritional Status. Nutr. Hosp..

[3] García-Sánchez C., Navarro R.M., Karcher C., de la Rubia A. (2023). Physical Demands during Official Competitions in Elite Handball: A Systematic Review. Int. J. Environ. Res. Public. Health.

[4] Janiczak A., Alcock R., Forsyth A., Trakman G.L. (2024). A Systematic Review of Interventions Targeting Modifiable Factors That Impact Dietary Intake in Athletes. Br. J. Nutr..

[5] Thomas D.T., Erdman K.A., Burke L.M. (2016). Nutrition and Athletic Performance. Med. Sci. Sports Exerc..

[6] Castillo M., Lozano-Casanova M., Sospedra I., Norte A., Gutiérrez-Hervás A., Martínez-Sanz J.M. (2022). Energy and Macronutrients Intake in Indoor Sport Team Athletes: Systematic Review. Nutrients.

[7] Kerksick C.M., Wilborn C.D., Roberts M.D., Smith-Ryan A., Kleiner S.M., Jäger R., Collins R., Cooke M., Davis J.N., Galvan E. (2018). ISSN Exercise & Sports Nutrition Review Update: Research & Recommendations. J. Int. Soc. Sports Nutr..

[8] Aragon A.A., Schoenfeld B.J., Wildman R., Kleiner S., VanDusseldorp T., Taylor L., Earnest C.P., Arciero P.J., Wilborn C., Kalman D.S. (2017). International Society of Sports Nutrition Position Stand: Diets and Body Composition. J. Int. Soc. Sports Nutr..

[9] Muñoz A., López-Samanes Á., Domínguez R., Moreno-Pérez V., Jesús Sánchez-Oliver A., Del Coso J. (2020). Use of Sports Supplements in Competitive Handball Players: Sex and Competitive Level Differences. Nutrients.

[10] Vázquez-Espino K., Rodas-Font G., Farran-Codina A. (2022). Sport Nutrition Knowledge, Attitudes, Sources of Information, and Dietary Habits of Sport-Team Athletes. Nutrients.

[11] Heaney S., O’Connor H., Michael S., Gifford J., Naughton G. (2011). Nutrition Knowledge in Athletes: A Systematic Review. Int. J. Sport. Nutr. Exerc. Metab..

[12] Citarella R., Itani L., Intini V., Zucchinali G., Scevaroli S., Kreidieh D., Tannir H., El Masri D., El Ghoch M. (2019). Nutritional Knowledge and Dietary Practice in Elite 24-Hour Ultramarathon Runners: A Brief Report. Sports.

[13] Werner E.N., Guadagni A.J., Pivarnik J.M. (2022). Assessment of Nutrition Knowledge in Division I College Athletes. J. Am. Coll. Health.

[14] Klein D.J., Eck K.M., Walker A.J., Pellegrino J.K., Freidenreich D.J. (2021). Assessment of Sport Nutrition Knowledge, Dietary Practices, and Sources of Nutrition Information in NCAA Division III Collegiate Athletes. Nutrients.

[15] Abbey E.L., Wright C.J., Kirkpatrick C.M. (2017). Nutrition Practices and Knowledge among NCAA Division III Football Players. J. Int. Soc. Sports Nutr..

[16] Jenner S.L., Buckley G.L., Belski R., Devlin B.L., Forsyth A.K. (2019). Dietary Intakes of Professional and Semi-Professional Team Sport Athletes Do Not Meet Sport Nutrition Recommendations—A Systematic Literature Review. Nutrients.

[17] McKay A.K.A., Stellingwerff T., Smith E.S., Martin D.T., Mujika I., Goosey-Tolfrey V.L., Sheppard J., Burke L.M. (2022). Defining Training and Performance Caliber: A Participant Classification Framework. Int. J. Sports Physiol. Perform..

[18] Trakman G., Forsyth A., Devlin B., Belski R. (2016). A Systematic Review of Athletes’ and Coaches’ Nutrition Knowledge and Reflections on the Quality of Current Nutrition Knowledge Measures. Nutrients.

[19] Jackson A.S., Pollock M.L. (1985). Practical Assessment of Body Composition. Phys. Sportsmed..

[20] Cunningham J.J. (1980). A Reanalysis of the Factors Influencing Basal Metabolic Rate in Normal Adults. Am. J. Clin. Nutr..

[21] Trakman G.L., Brown F., Forsyth A., Belski R. (2019). Modifications to the Nutrition for Sport Knowledge Questionnaire (NSQK) and Abridged Nutrition for Sport Knowledge Questionnaire (ANSKQ). J. Int. Soc. Sports Nutr..

[22] Trakman G.L., Forsyth A., Hoye R., Belski R. (2018). Development and Validation of a Brief General and Sports Nutrition Knowledge Questionnaire and Assessment of Athletes’ Nutrition Knowledge. J. Int. Soc. Sports Nutr..

[23] Hector A.J., Phillips S.M. (2018). Protein Recommendations for Weight Loss in Elite Athletes: A Focus on Body Composition and Performance. Int. J. Sport. Nutr. Exerc. Metab..

[24] Jäger R., Kerksick C.M., Campbell B.I., Cribb P.J., Wells S.D., Skwiat T.M., Purpura M., Ziegenfuss T.N., Ferrando A.A., Arent S.M. (2017). International Society of Sports Nutrition Position Stand: Protein and Exercise. J. Int. Soc. Sports Nutr..

[25] Institute of Medicine (US) Food and Nutrition Board (1998). Dietary Reference Intakes.

[26] Danh J.P., Nucci A., Andrew Doyle J., Feresin R.G. (2023). Assessment of Sports Nutrition Knowledge, Dietary Intake, and Nutrition Information Source in Female Collegiate Athletes: A Descriptive Feasibility Study. J. Am. Coll. Health.

[27] Coapstick G.-J.A., Barry A.M., Levesque C.L., Shoemaker M.E. (2024). Nutrient Intake, Performance, and Body Composition of Preseason Wrestlers. Int. J. Exerc. Sci..

[28] Bettonviel A.E.O., Brinkmans N.Y.J., Russcher K., Wardenaar F.C., Witard O.C. (2016). Nutritional Status and Daytime Pattern of Protein Intake on Match, Post-Match, Rest and Training Days in Senior Professional and Youth Elite Soccer Players. Int. J. Sport. Nutr. Exerc. Metab..

[29] Andrews M.C., Itsiopoulos C. (2016). Room for Improvement in Nutrition Knowledge and Dietary Intake of Male Football (Soccer) Players in Australia. Int. J. Sport. Nutr. Exerc. Metab..

[30] Anderson L., Orme P., Naughton R.J., Close G.L., Milsom J., Rydings D., O’Boyle A., Di Michele R., Louis J., Hambly C. (2017). Energy Intake and Expenditure of Professional Soccer Players of the English Premier League: Evidence of Carbohydrate Periodization. Int. J. Sport. Nutr. Exerc. Metab..

[31] Han Q., Xiang M., An N., Tan Q., Shao J., Wang Q. (2024). Effects of Vitamin D3 Supplementation on Strength of Lower and Upper Extremities in Athletes: An Updated Systematic Review and Meta-Analysis of Randomized Controlled Trials. Front. Nutr..

[32] Tomlinson P.B., Joseph C., Angioi M. (2015). Effects of Vitamin D Supplementation on Upper and Lower Body Muscle Strength Levels in Healthy Individuals. A Systematic Review with Meta-Analysis. J. Sci. Med. Sport..

[33] Elsahoryi N.A., Trakman G., Al Kilani A. (2021). General and Sports Nutrition Knowledge among Jordanian Adult Coaches and Athletes: A Cross-Sectional Survey. PLoS ONE.

[34] Magee M.K., Jones M.T., Fields J.B., Kresta J., Khurelbaatar C., Dodge C., Merfeld B., Ambrosius A., Carpenter M., Jagim A.R. (2023). Body Composition, Energy Availability, Risk of Eating Disorder, and Sport Nutrition Knowledge in Young Athletes. Nutrients.

[35] Torres-McGehee T.M., Pritchett K.L., Zippel D., Minton D.M., Cellamare A., Sibilia M. (2012). Sports Nutrition Knowledge Among Collegiate Athletes, Coaches, Athletic Trainers, and Strength and Conditioning Specialists. J. Athl. Train..

[36] Moncef C., Said M., Olfa N., Dagbaji G. (2012). Influence of Morphological Characteristics on Physical and Physiological Performances of Tunisian Elite Male Handball Players. Asian J. Sports Med..

[37] Chaouachi A., Brughelli M., Levin G., Boudhina N.B.B., Cronin J., Chamari K. (2009). Anthropometric, Physiological and Performance Characteristics of Elite Team-Handball Players. J. Sports Sci..

[38] Jonvik K.L., King M., Rollo I., Stellingwerff T., Pitsiladis Y. (2022). New Opportunities to Advance the Field of Sports Nutrition. Front. Sports Act. Living.

